# Flagellin/TLR5 Stimulate Myeloid Progenitors to Enter Lung Tissue and to Locally Differentiate Into Macrophages

**DOI:** 10.3389/fimmu.2021.621665

**Published:** 2021-03-19

**Authors:** Xin Lei, Jara Palomero, Iris de Rink, Tom de Wit, Martijn van Baalen, Yanling Xiao, Jannie Borst

**Affiliations:** ^1^Division of Tumor Biology and Immunology, The Netherlands Cancer Institute, Amsterdam, Netherlands; ^2^Department of Immunology and Oncode Institute, Leiden University Medical Center, Leiden, Netherlands; ^3^Genomics Facility, The Netherlands Cancer Institute, Amsterdam, Netherlands; ^4^Flow Cytometry Facility, The Netherlands Cancer Institute, Amsterdam, Netherlands

**Keywords:** Flagellin, TLR5, myelopoiesis, macrophage, epithelium, CCR2

## Abstract

Toll-like receptor 5 (TLR5) is the receptor of bacterial Flagellin. Reportedly, TLR5 engagement helps to combat infections, especially at mucosal sites, by evoking responses from epithelial cells and immune cells. Here we report that TLR5 is expressed on a previously defined bipotent progenitor of macrophages (MΦs) and osteoclasts (OCs) that resides in the mouse bone marrow (BM) and circulates at low frequency in the blood. *In vitro*, Flagellin promoted the generation of MΦs, but not OCs from this progenitor. *In vivo*, MΦ/OC progenitors were recruited from the blood into the lung upon intranasal inoculation of Flagellin, where they rapidly differentiated into MΦs. Recruitment of the MΦ/OC progenitors into the lung was likely promoted by the CCL2/CCR2 axis, since the progenitors expressed CCR2 and type 2 alveolar epithelial cells (AECs) produced CCL2 upon stimulation by Flagellin. Moreover, CCR2 blockade reduced migration of the MΦ/OC progenitors toward lung lavage fluid (LLF) from Flagellin-inoculated mice. Our study points to a novel role of the Flagellin/TLR5 axis in recruiting circulating MΦ/OC progenitors into infected tissue and stimulating these progenitors to locally differentiate into MΦs. The progenitor pathway to produce MΦs may act, next to monocyte recruitment, to fortify host protection against bacterial infection at mucosal sites.

## Introduction

Epithelial cells are the first line of defense against infection (1, 2). Tissue resident myeloid cells contribute to defense once epithelial integrity is broken. At steady state, peripheral tissues harbor resident MΦs that have an embryonic origin and migratory dendritic cells (DCs) that are constantly generated from hematopoietic progenitors in the BM (3, 4). Upon infection or tissue damage, neutrophils and monocytes are recruited from the blood into peripheral tissues (5). All myeloid cell types have pattern recognition receptors (PRRs), such as toll-like receptors (TLRs) that allow them to recognize and respond to pathogen-associated molecular patterns (PAMPs) and self-derived damage-associated molecular patterns (DAMPs) (6).

TLR5 recognizes Flagellin, the primary structural component of bacterial flagella that is a major trigger of innate immunity (7, 8), particularly at mucosal sites (9, 10). TLR5 is located at the basal side of epithelia and thereby senses the invasion of potentially pathogenic bacteria, such as Salmonella (11, 12). In epithelial cells, Flagellin induces expression of proinflammatory cytokines, chemokines and nitric oxide (13). Myeloid cell types as well as T cells also respond to Flagellin (10, 14, 15). In the mouse intestine, TLR5 on CD11c^+^ cells in the lamina propria proved to be essential for the defense against pathogenic bacteria (16). Flagellin is a key antigen in human Crohn's disease (17), supporting its role in evoking both innate and adaptive immunity. Accordingly, many studies address the potency of Flagellin as a vaccine adjuvant (18).

TLRs are also found on different hematopoietic stem and progenitor cells (HSPCs), including hematopoietic stem cells (HSCs), common myeloid progenitors (CMPs), and granulocyte (G)/MΦ progenitors (GMPs). TLR signaling preferentially stimulates myeloid development from HSPCs in the absence of homeostatic cytokines. This suggests a role for TLR signaling in innate immune cell replenishment (19–21). Recently, it has been reported that systemic Flagellin administration drives rapid TLR5-dependent HSC cell proliferation and a great increase in multipotent progenitors. Neutrophils produced by these progenitors protected mice against total body irradiation (22). The generation of myeloid cells from hematopoietic progenitors fits in the concept of “emergency hematopoiesis.” Herein, myeloid cells are generated *de novo* from progenitors, to boost immune defense and tissue repair (23).

OCs are also part of the myeloid lineage (24–26) and play a role in hematopoiesis by creating niches for stem cells in the BM (27). OCs and osteoblasts maintain bone homeostasis *via* bidirectional communication. This process is deregulated during chronic inflammation, such as in rheumatoid arthritis, leading to bone remodeling (28, 29). We previously found that the GMP as found in mouse and human BM can also give rise to OCs and DCs. We thus redefined the GMP as oligopotent G/MΦ/OC/DC progenitor (GMODP). In addition, we found that the GMODP stepwise gives rise to a more committed MΦ/OC/DC progenitor (MODP) and a bipotent MΦ/OC progenitor (MOP) (25, 26). We here report that TLR5 cell surface expression progressively increases with MΦ/OC commitment in this hierarchy and that Flagellin/TLR5 promotes MΦ- but not OC differentiation from MOPs. Furthermore, our study supports a scenario wherein TLR5 on bipotent MΦ/OC myeloid progenitors helps replenish MΦs for protection against bacterial infection at mucosal sites.

## Materials and Methods

### Mice

Wild-type (WT) CD45.1 and CD45.2 C57BL/6 mice of 6–12 weeks of age were used for *in vitro* or *in vivo* experiments. For *in vivo* experiments, age-matched female mice were used. For *in vitro* and *ex vivo* experiments, male and female mice were used at random. Experiments were performed in accordance with national guidelines and approved by the Experimental Animal Committee of The Netherlands Cancer Institute.

### Isolation of BM Mononuclear Cells

Tibiae, femurs and ilia were washed with phosphate buffered saline (PBS) and ground in a mortar in presence of PBS. BM cells were flushed out of the bone fragments with RPMI-1640 medium (Gibco, Life Technologies) with 10% fetal calf serum (FCS) and the cell suspension was forced through a 70 μm cell strainer to remove bone chips and other debris. Cells were recovered by centrifugation and erythrocytes were removed with eBioscience™ red blood cell lysis buffer. After incubation on ice for 1–2 min, RPMI-1640 medium with 10% FCS was added and BM cells were collected by centrifugation and resuspended in medium until further use.

### Intranasal Flagellin Administration and Adoptive Cell Transfer

MOPs from pooled BM of CD45.1 female donor mice (*n* = 5) were flow cytometrically sorted and transferred intravenously (i.v.) *via* the retro-orbital plexus into age-matched female CD45.2 recipient mice (*n* = 8) at 100,000 cells in 200 μl PBS per mouse. At 24 h after adoptive transfer, recipient mice were anesthetized by methoxyflurane inhalation, and intranasally (i.n.) inoculated with 1 μg Flagellin (Invitrogen) in 50 μl PBS per mouse (*n* = 4) or with 50 μl PBS (*n* = 4).

### Lung Lavage Fluid

Mice were i.n. inoculated with 1 μg Flagellin in 50 μl PBS (*n* = 4) or PBS only (*n* = 3). At 3–4 h after inoculation, mice were sacrificed and lungs together with intact tracheae were harvested. 200 μl PBS was injected into the trachea with a 1 ml syringe with a blunted 25 gauge needle until all lobes of the lung were bloated. The lung was flushed three times with the PBS while the needle was fixed in the trachea. Next, the liquid in the syringe was harvested as LLF.

### Cell Isolation From Lung for CCL2 Detection

Lungs were minced into small pieces (<0.5 mm^3^) and incubated in 5 ml of 200 mg/ml Liberase TL Research Grade (Sigma-Aldrich) at 37°C for 15 min. Cells were liberated from tissue by continuously pipetting the sample for 15 min at room temperature. Cells were filtered through a 40 μm strainer (BD Biosciences) and washed twice with RPMI-1640 containing 0.01% deoxyribonuclease I (DNase, Sigma-Aldrich). After red blood cell lysis, cells were plated in non-adherent round-bottom 96-well plates (BD Falcon) at a density of 300,000 cells/well and subsequently treated with or without Flagellin (100 ng/ml) in RPMI-1640 medium with 1% FCS. Protein transport inhibitor (GolgiPlug, BD Biosciences) was added to the cultures at a concentration of 1:1,000 and 12 h later, cells were harvested, stained for CD45, MHC-II, CD31, and CCL2 and analyzed by flow cytometry.

### Flow Cytometry

For isolation of MΦ/OC progenitor cell populations, mouse BM cells were incubated with the identifying antibodies (Supplementary Table 1) in cell staining buffer (Biolegend) for 45 min on ice. Dead cells were excluded by staining with 7-amino-actinomycin D (7-AAD) or 4,6-Diamidino-2-Phenylindole (DAPI). In order to prevent clump formation from dead cells, 0.01% DNase was added. Cell sorting was performed on BD FACSAriaTM Fusion (BD Biosciences). For analysis, *ex vivo* cells and *in vitro* cultured cells in mouse experiments were stained with indicated antibodies (Supplementary Table 1) in cell staining buffer for 45 min on ice and analyzed on a BD™ LSR II SORP, BD LSRFortessa™, or BD FACSymphony™ A5 SORP flow cytometer (BD Biosciences). Fluorescence minus one (FMO) was used as negative control where indicated. Data were analyzed with FlowJo™ software (BD). The gating strategy for identifying MODP and MOP populations in relation to other HSC populations is shown in Supplementary Figure 1. The same gating strategy has been used throughout this study to identify MODP and MOP populations. For analysis of all other cell populations, gating strategies are provided in the figures. For detection of c-Fms (CD115) cell surface expression, we originally used direct *ex vivo* staining, but later preincubated the BM cells overnight in culture medium at 37°C, 5% CO_2_ prior to staining, which enhanced the signal (Supplementary Figures 1, 4, 9).

### *In vitro* Differentiation of Mouse Progenitors Into OC, MΦ, and DC

MODP and MOP cell populations were sorted by flow cytometry and seeded in 96-well plates (BD Falcon) (flat bottom for OC differentiation, round bottom for MΦ and DC differentiation) at 2,000–3,000 cells/well. To induce OC differentiation, progenitor cells were cultured in a minimum essential medium (α-MEM; Gibco, Life Technologies) with 10% FCS, supplemented with 20 ng/ml recombinant mouse (rm) RANK ligand (RANKL, R&D) and 25 ng/ml rm macrophage colony-stimulating factor (M-CSF) (Peprotech). To induce MΦ differentiation, progenitor cells were cultured in IMDM with 10% FCS, supplemented with 25 ng/ml rm M-CSF. To induce DC differentiation, progenitor cells were cultured in IMDM with 10% FCS, supplemented with 200 ng/ml rm FLT3 ligand (R&D). Flagellin (0.5 μg/ml; Invitrogen) was added into cultures as specified. OC differentiation was assessed by tartrate-resistant acid phosphatase (TRAP) staining (Sigma-Aldrich) according to manufacturer's instructions after 6 days of culture. Cells were washed with PBS, fixed with paraformaldehyde (PFA; Sigma-Aldrich) for 15 min at RT. Fixed cells were washed twice with pre-warmed water to remove remaining PFA. Next, cells were incubated with TRAP staining solution for 5–10 min at RT and washed according to manufacturer's instructions. TRAP^+^ OCs were analyzed by light microscopy (Nikon) and quantified using ImageJ (National Institutes of Health).

### *In vitro* Transwell Migration Assay

Total BM cells or sorted progenitors were plated at equal numbers (100,000 cells per well or 5,000 cells per well, respectively) in the upper chamber of a 24-well or a 96-well HTS 3 μm polycarbonate transwell plate (Corning) in 100 μl serum-free IMDM (Gibco, Life Technologies). Sorted progenitors were seeded in the presence or absence of CCR2 blocking mAb (0.75 μM; Tocris). The lower chambers contained 300 μl LLF from PBS-, or Flagellin-treated mice. After 12 h, cells that had migrated into lower chambers were collected, stained with antibodies and analyzed by flow cytometry.

### Statistical Analysis

Statistical evaluation was performed in GraphPad Prism 8 (GraphPad Software, Inc.) using unpaired two-tailed Student's *t-*test or one way ANOVA test.

## Results

### TLR5 Is Differentially Expressed Between MODPs and MOPs

**Figure 1:**
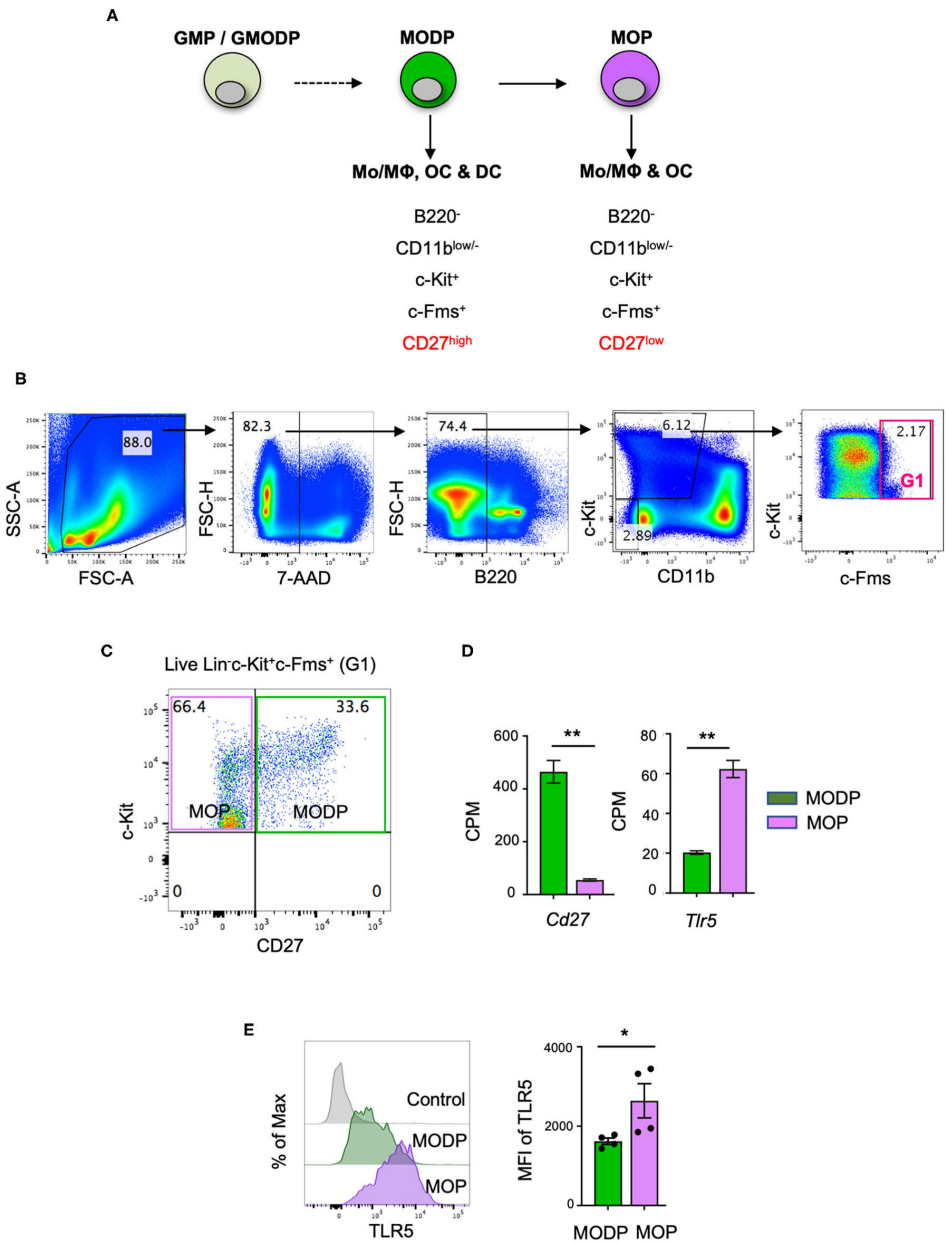
TLR5 is differentially expressed at transcript and protein levels between MODPs and MOPs. **(A)** Proposed developmental relationship between GMP/GMODP, MODP, and MOP in murine hematopoiesis and the identifying cell surface markers (24, 26). **(B)** Representative flow cytometry plots depicting gating strategy for B220^−^CD11b^low/−^c-Kit^+^c-Fms^+^ BM cells. **(C)** Representative flow cytometry plot depicting how CD27 cell surface expression discriminates MODPs and MOPs within the B220^−^CD11b^low/−^c-Kit^+^c-Fms^+^ BM cell population. **(D)** Data from an earlier study in which BM-derived MODPs and MOPs were analyzed by RNAseq (GSE97380). *Cd27* and *Tlr5* mRNA levels depicted are based on normalized read counts (counts per million, CPM) (*n* = 3, cells pooled from 2 mice per sample) (26). **(E)** Flow cytometric analysis of TLR5 cell surface expression on MODP and MOP populations. TLR5 FMO was used as control, MFI = median fluorescence intensity. Data are pooled from 2 independent experiments, each with cells from 2 mice. Error bars indicate standard error of the mean (SEM) and unpaired two tailed Student's *t*-test was used for statistical evaluation (*p < 0.05, **p < 0.01).

In a previous study, we have redefined the human GMP as oligopotent progenitor of G, MΦ, OC, and DC and termed it GMODP. Downstream of it, we identified a more committed oligopotent progenitor of MΦ, OC, and DC (MODP) (25) (Figure 1A). In the mouse, we identified common progenitors of MΦ, OC, and DC in a B220^−^CD11b^low/−^c-Kit^+^c-Fms^+^ BM cell population (Figure 1B). This population could be dissected into a CD27^high^ MODP and a downstream CD27^low/−^ MΦ/OC progenitor (MOP) (26) (Figures 1A,C). We analyzed in one staining procedure the cell surface phenotypes of MODP and MOP populations and the main hematopoietic progenitor populations HSC, multipotent progenitor (MPP), common lymphoid progenitor (CLP), CMP, and GMP, as defined by Weissman and Shizuru (30). This analysis shows that the MODP and MOP populations are distinct from their upstream progenitors, including the GMP (Supplementary Figures 1A–C). The developmental relationship between MODP and MOP was further validated by mRNA deep sequencing (26). Among transcripts that discriminated between MODP and MOP were *Cd27* (*Tnfrsf7*), but also *Tlr5* (Figure 1D). TLR5 was also differentially expressed between the two progenitor cell populations at the protein level, as determined by flow cytometry. The cell surface expression of TLR5 was significantly higher on MOP than on MODP cells that occur in low frequency in the mouse BM (Figure 1E, Supplementary Figures 2A,B).

### TLR5 Cell Surface Level Correlates With MOP Commitment to MΦ/OC Differentiation

**Figure 2:**
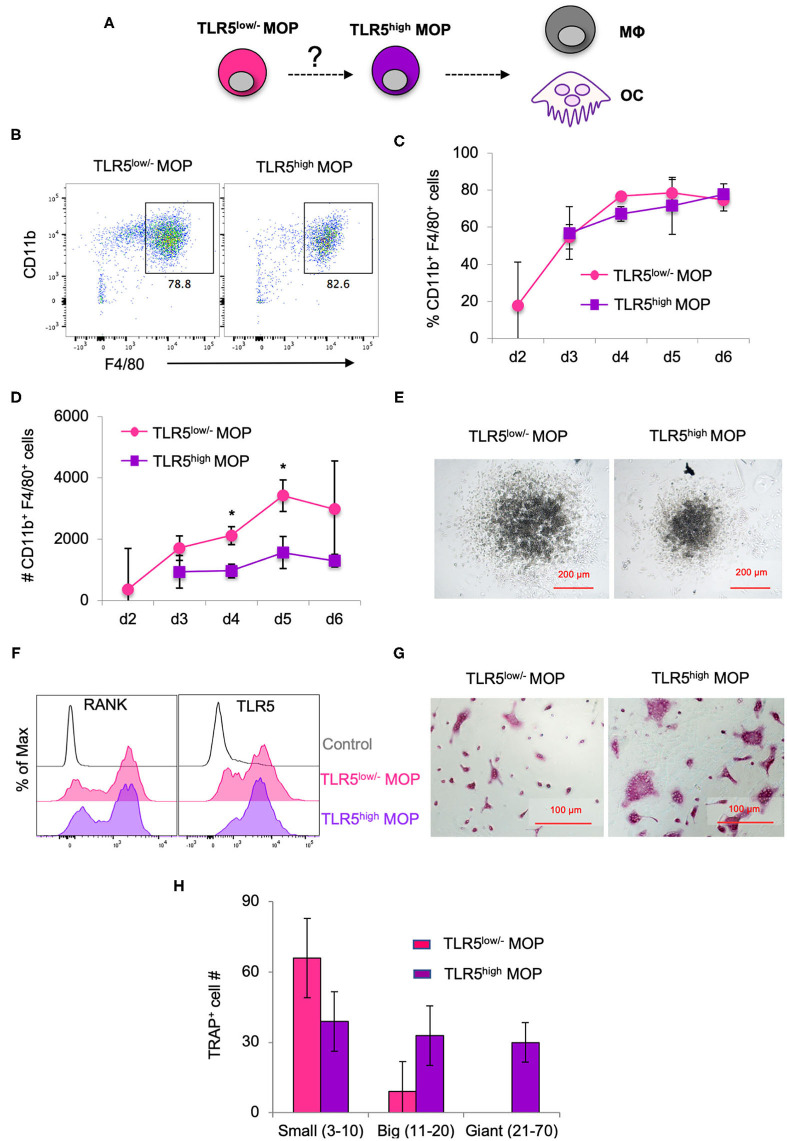
MΦ and OC differentiation potential of TLR5^low/−^ MOPs and TLR5^high^ MOPs. **(A)** Proposed developmental path from MOP to MΦ and OC. **(B–H)** MΦ and OC differentiation cultures. TLR5^low/−^ and TLR5^high^ MOPs were flow cytometrically sorted from pooled BM cells of 3–5 mice, plated at 2,000 cells/well and cultured under MΦ- (M-CSF) **(B–E)** or OC (M-CSF + RANKL) **(F–H)** differentiation conditions. **(B)** Representative flow cytometry plots identifying MΦs as MHCII^+^CD11b^+^F4/80^+^ cells in MΦ differentiation cultures at day 5. **(C,D)** Frequencies (%) **(C)** and absolute numbers (#) **(D)** of MHCII^+^CD11b^+^F4/80^+^ cells in MΦ differentiation cultures as determined by flow cytometry at days 2–6. Data are pooled from three independent experiments with technical duplicate samples. **(E)** Representative microscopic images of the MΦ differentiation cultures at day 5. **(F)** Representative RANK and TLR5 cell surface expression on cells in OC differentiation cultures at day 3. FMO controls were used for RANK and TLR5 staining. **(G)** Representative light microscopic images depicting TRAP-stained cells in OC differentiation cultures at day 6. **(H)** Absolute numbers of TRAP^+^ cells (OC) in OC differentiation cultures at day 6. Cells with multiple nuclei were counted as individual cell and discriminated into small (1–10 nuclei), big (11–20 nuclei) and giant OCs (≥21 nuclei). Data are pooled from two independent experiments, each with technical triplicate samples. Error bars indicate SEM and unpaired two tailed Student's *t*-test was used for statistical evaluation (*p < 0.05).

Given that TLR5 cell surface levels on the MOP population were heterogeneous and higher than on the MODP population, we examined whether they correlated with commitment to MΦ and OC differentiation (Figure 2A). For this purpose, TLR5^low/−^ and TLR5^high^ MOP cell subsets were purified from BM cells by flow cytometry-based sorting and *in vitro* differentiation cultures were performed. To determine differentiation into MΦs, defined as MHCII^+^CD11b^+^F4/80^+^ cells (Figure 2B), progenitor populations were cultured at 2,000 cells per well with M-CSF and analyzed by flow cytometry at successive days until day 6. TLR5^low/−^ and TLR5^high^ MOP-derived cultures had comparable frequencies of MΦs, throughout the entire duration of the culture (Figure 2C). However, the MΦ yield in absolute cell numbers was higher in the TLR5^low/−^ MOP-derived cultures than in TLR5^high^ MOP-derived cultures (Figure 2D). This suggests that TLR5^high^ MOPs are more committed and less proliferative, as also appeared from microscopic examination (Figure 2E). In agreement with the notion that TLR5 expression levels correlated with MΦ commitment, TLR5 expression was higher on MHCII^+^CD11b^+^F4/80^+^ MΦs than on immature MHCII^+^CD11b^low/−^F4/80^−^ cells in a progenitor-derived MΦ differentiation culture (Supplementary Figures 2C,D).

To determine differentiation into OCs, TLR5^low/−^ and TLR5^high^ MOPs were cultured at 2,000 cells per well with RANKL and M-CSF. At day 3 of culture, RANK was expressed on a large part of the cells in both cultures (Figure 2F). OCs are characterized by expression of TRAP and during maturation, single cells fuse to form multinucleated OCs of different sizes (31). At day 6 of culture, OC formation was diagnosed microscopically and quantified by TRAP staining. TLR5^high^ MOP-derived OCs had the most mature phenotype, with giant multinucleated TRAP^+^ cells that were not seen in TLR5^low/−^ MOP-derived offspring (Figures 2G,H), indicating that TLR5^high^ MOPs are more committed to OC differentiation than TLR5^low/−^ MOPs. Overall, we conclude that cell surface TLR5 levels on the MOP population positively correlate with commitment to MΦ/OC differentiation.

### Flagellin Promotes MΦ Differentiation From MODPs and MOPs *in vitro*

**Figure 3:**
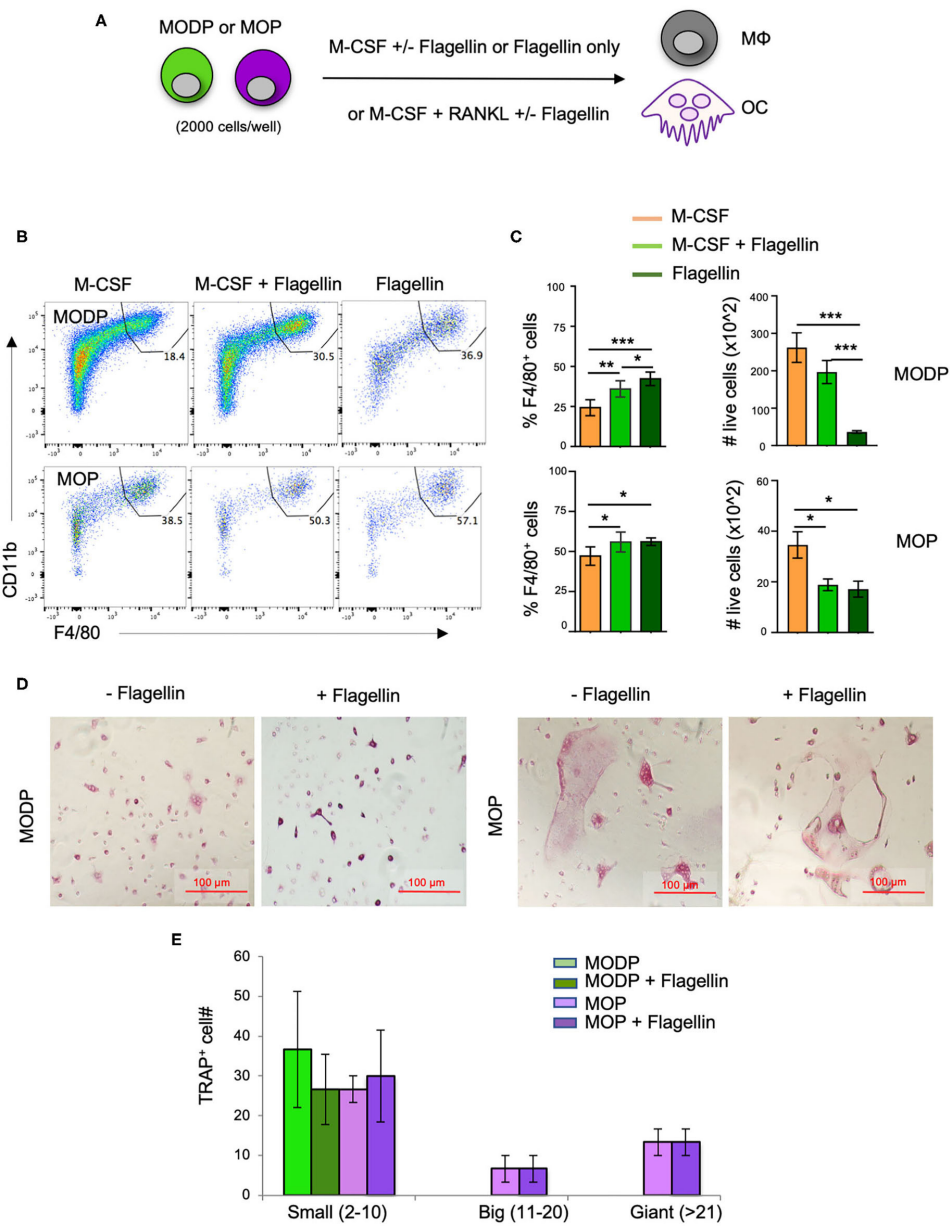
Flagellin promotes MΦ and OC differentiation from progenitors *in vitro*. **(A)** Schematic representation of the differentiation cultures. MODPs and MOPs were flow cytometrically sorted from pooled BM cells of 3–5 mice, plated at 2,000 cells/well and cultured under MΦ- (M-CSF; **B,C**) or OC differentiation conditions (M-CSF + RANKL; **D,E**) with or without Flagellin. **(B)** Representative flow cytometry plots depicting the frequencies (%) of MΦs, defined as MHCII^+^CD11b^+^F4/80^+^ cells in MΦ differentiation cultures at day 3. **(C)** Quantification of MΦ frequencies and total MΦ cell numbers in the MΦ differentiation cultures. Data are pooled from three independent experiments, each with technical duplicate samples. **(D)** Representative light microscopic images of cells in OC differentiation cultures. **(E)** Quantitative analysis of OC offspring from progenitors under indicated conditions. OC yield was quantified as outlined in the legend of Figure 2H. Data are pooled from two independent experiments, each with technical duplicate samples. Error bars indicate SEM and one way ANOVA test and Tukey's multiple comparison test were used for statistical evaluation (*p < 0.05, **p < 0.01, ***p < 0.001).

Next, we investigated whether TLR5 signals impacted MΦ- and/or OC differentiation from MODPs and MOPs. For this purpose, we added Flagellin to the differentiation cultures (Figure 3A). In presence of M-CSF, both MODP and MOP cells differentiated into MΦs (Figure 3B). Within 1 day of differentiation, already 30–40% of MOP offspring cells had the MHCII^+^CD11b^+^F4/80^+^ MΦ phenotype (Supplementary Figures 3A,B), which increased to 50–60% at day 3 (Figure 3B, lower panel). Addition of Flagellin to M-CSF increased the frequency of MΦs both in MODP- and MOP-derived cultures, as compared to culture with M-CSF alone (Figures 3B,C). Flagellin alone could also induce MΦ differentiation from the progenitors (Figures 3B,C). However, live cell yield was significantly lower when Flagellin was present, either alone or in combination with M-CSF (Figure 3C). To confirm that Flagellin was driving MΦ differentiation *via* TLR5, we performed MΦ differentiation cultures with BM cells from *Tlr5*^+/+^ and *Tlr5*^−*/*−^ littermate mice. Cell surface staining validated TLR5 expression on *Tlr5*^+/+^ Lin^−^c-Kit^+^c-Fms^+^ BM cells and lack of it on the same cell population from *Tlr5*^−*/*−^ mice (Supplementary Figures 4A,B). In *Tlr5*^+/+^ BM cell cultures the percentage of MΦs increased upon stimulation with M-CSF or Flagellin as compared to the untreated control sample. In contrast, in *Tlr5*^−*/*−^ BM cell cultures the percentage of MΦs increased only upon stimulation with M-CSF but not Flagellin (Supplementary Figures 4C–E). These results indicate that direct sensing of Flagellin by TLR5 on the progenitors promoted their differentiation into MΦs.

To test the impact of Flagellin on OC differentiation, progenitors were cultured with RANKL and M-CSF and offspring cells were sampled at day 6 of culture. Both MODPs and MOPs differentiated into OCs, defined as multinucleated TRAP^+^ cells (Figure 3D), as previously described (26). Additional presence of Flagellin did not affect number and maturity of OC offspring from MODP or MOP, as diagnosed by microscopy and TRAP^+^ cell quantification (Figure 3E). In summary, we found that *in vitro*, TLR5 engagement on MODP and MOP by Flagellin promotes MΦ differentiation, but does not influence OC differentiation.

### Flagellin Promotes Recruitment of MOPs Into Lung Tissue and Their Local Differentiation Into MΦs

**Figure 4:**
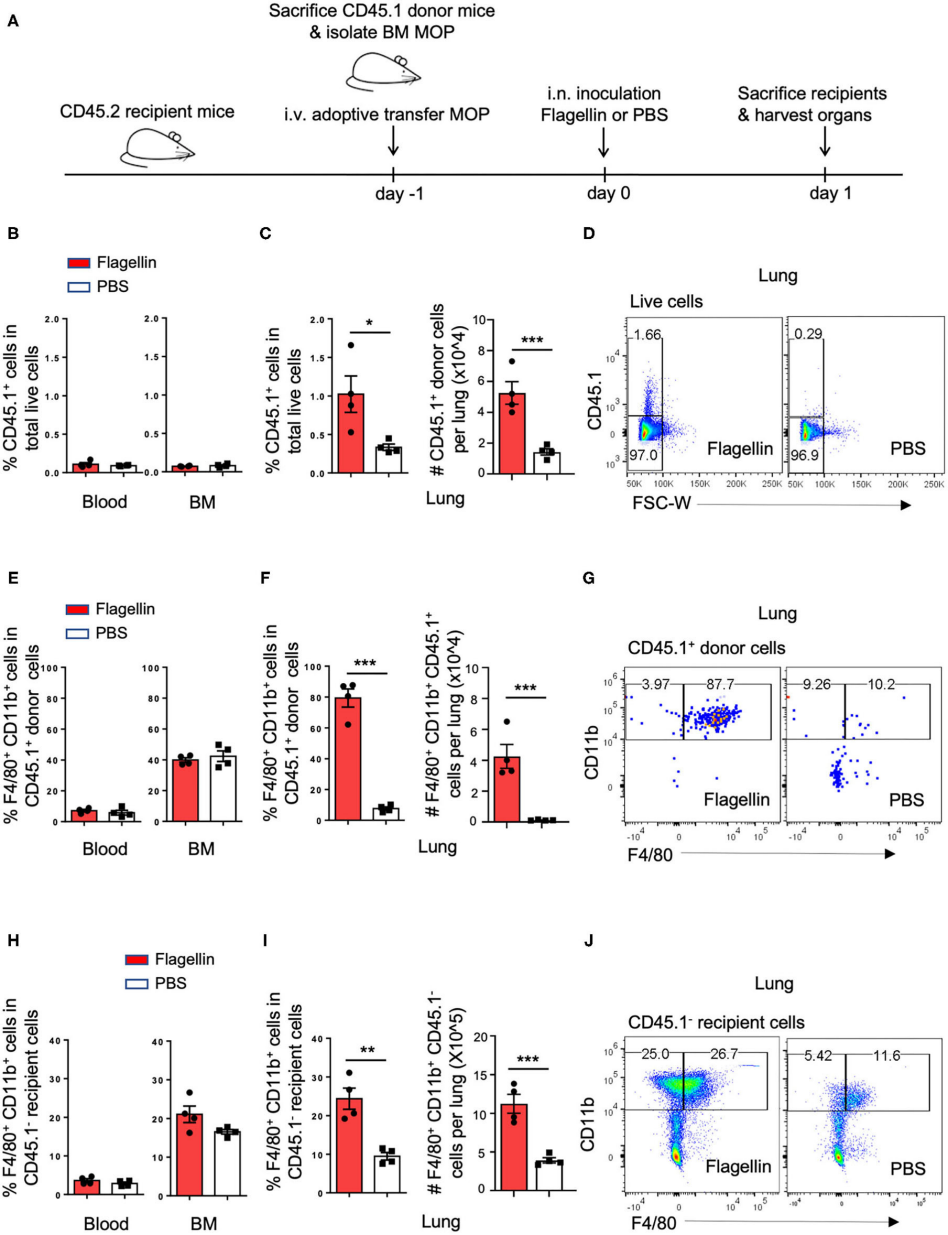
I.n. Flagellin inoculation promotes MΦ differentiation from MOPs in the lung. **(A)** Schematic representation of work flow: MOPs were flow cytometrically sorted from pooled BM cells of CD45.1 donor mice (*n* = 5) and transferred i.v. into CD45.2 recipient mice (*n* = 8). At 24 h after adoptive cell transfer, 1 μg Flagellin in 50 μl PBS or 50 μl PBS only was inoculated i.n. into the recipient mice (*n* = 4 for each group) and 24 h later, lung, blood and BM were harvested and dissociated into single cells for flow cytometric analysis. **(B,E,H)** Quantification of CD45.1^+^ cells, CD45.1^+^ MOP-derived MΦs and CD45.1^−^ endogenous MΦs in the blood and BM, respectively, as indicated by frequencies (%). **(C,F,I)** Quantification of CD45.1^+^ cells, CD45.1^+^ MOP-derived MΦs and CD45.1^−^ endogenous MΦs in the lung, as indicated by frequencies (%) and absolute numbers (#). **(D,G,J)** Representative flow cytometric plots depicting adoptively transferred CD45.1^+^ cells, CD45.1^+^ MOP-derived MΦs and CD45.1^−^ endogenous MΦs in the lung, respectively. MΦs were defined as MHCII^+^CD11b^+^F4/80^+^ cells. Data are representative of two independent experiments. Error bars indicate SEM, and unpaired two tailed Student's *t-*test was used for statistical evaluation (*p < 0.05, **p < 0.01, ***p < 0.001).

Since Flagellin promoted MΦ differentiation from MODPs and MOPs *in vitro*, we hypothesized that Flagellin could also promote MΦ differentiation from myeloid progenitors *in vivo*. To test this, we used a mouse model of intranasal (i.n.) inoculation of Flagellin that reportedly prompts a MΦ response in the lung (32). CD45.1^+^ MOPs were transferred intravenously (i.v.) *via* the retro-orbital plexus into CD45.2 recipient mice (Figure 4A; Supplementary Figure 5). Flow cytometric settings for detection of CD45.1^+^ cells in lung, blood and BM of CD45.2 recipient mice were determined prior to adoptive cell transfer (Supplementary Figures 6A,B). Furthermore, we verified that flow-sorted CD45.1^+^ donor MOPs were able to differentiate into OCs and MΦs *in vitro*, prior to adoptive transfer (Supplementary Figure 6C).

We had two groups of CD45.2 mice that both received CD45.1^+^ MOPs and were stimulated 1 day later i.n. with either Flagellin or PBS. After 24 h, cells were isolated from lung, blood and BM of these mice and analyzed by flow cytometry (Figure 4A, Supplementary Figure 5). Strikingly, the frequency and total number of CD45.1^+^ donor cells were increased upon Flagellin stimulation in the lung, but not in blood or BM (Figures 4B–D). Likewise the frequency and total number of CD45.1^+^ MΦs were increased upon Flagellin stimulation in the lung, but not in blood or BM (Figures 4E–G; Supplementary Figures 6D,E,G,H). We also looked at the endogenous response to Flagellin. Frequency and total number of CD45.1^−^ recipient MΦs (Figures 4H–J; Supplementary Figures 6F,I) increased upon Flagellin stimulation in the lung, but not in blood or BM. We also detected an increase in CD45.1^−^ recipient MOPs in the lung after Flagellin stimulation (Supplementary Figures 7A,B). These results suggest that i.n. Flagellin administration recruitment of MOPs from the blood circulation into the lung and their local differentiation into MΦs.

### MOP Recruitment Into Lung Tissue Upon Flagellin Administration Relies on the CCL2-CCR2 Axis

**Figure 5:**
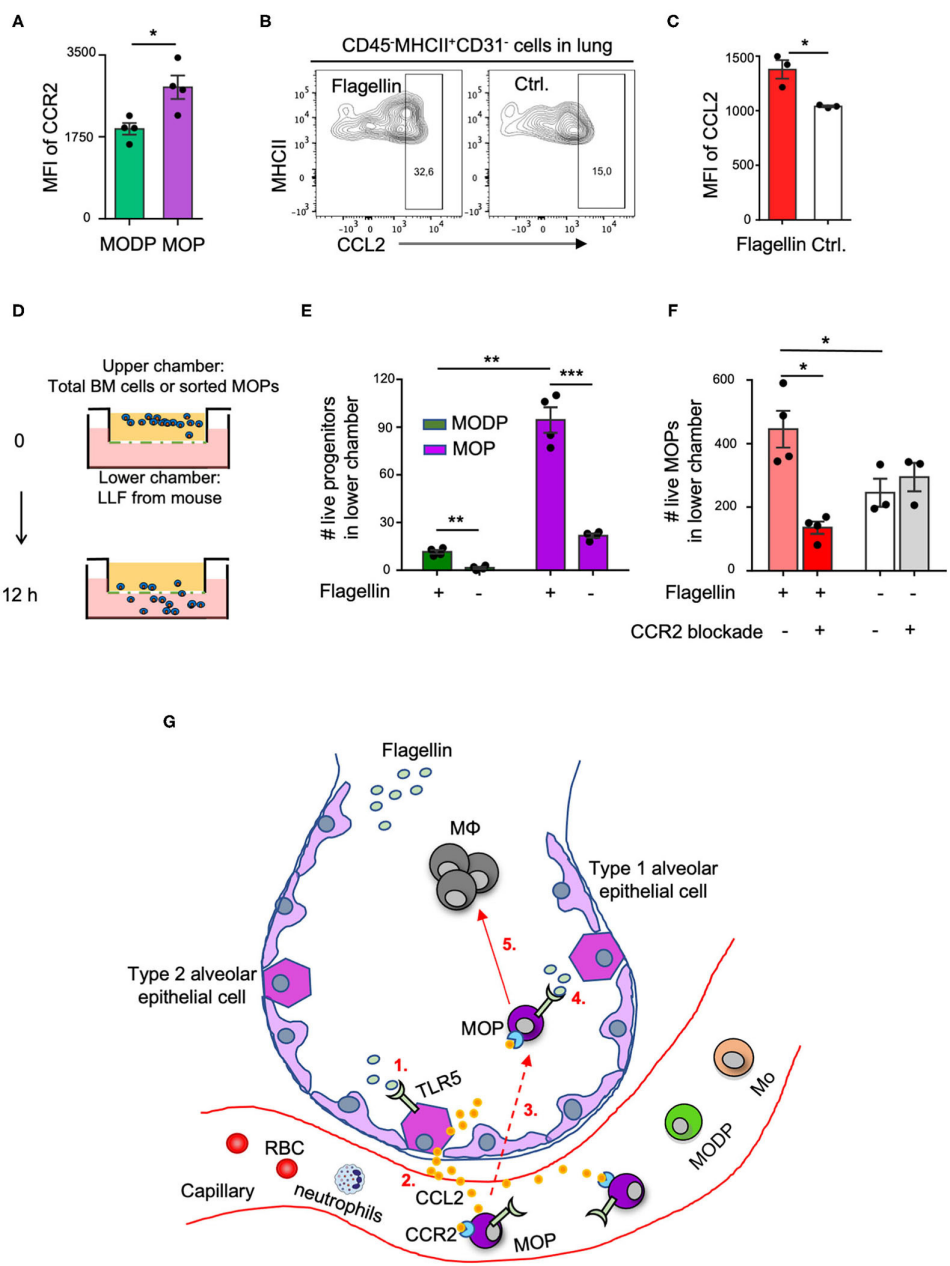
The CCL2-CCR2 axis promotes Flagellin-induced MOP migration into the lung. **(A)** CCR2 cell surface expression on MODPs and MOPs from BM (*n* = 4), as analyzed by flow cytometry (MFI, median fluorescence intensity). Data are representative of two independent experiments. **(B,C)** CCL2 expression in type 2 AECs, defined as CD45^−^MHCII^+^CD31^−^ cells, as determined by intracellular staining and flow cytometry in single-cell suspensions of lung tissue after overnight culture in medium with Flagellin or without (Ctrl.). **(B)** Representative flow cytometry plots depicting the frequencies (%) of CCL2^+^ cells within type 2 AECs. CCL2 FMO was used as control. **(C)** Representative quantitative data from one out of two independent experiments with pooled cells from 3 mice analyzed in technical triplicates. **(D–F)** Transwell migration assay with LLF. **(D)** Experimental set-up. Total BM cells (100,000 cells pooled from three mice) **(E)**, or sorted MOPs (5,000 cells pooled from three mice) **(F)** were added per upper well of a 24 well or 96 well plate and allowed to migrate toward LLF from the two groups of mice (*n* = 3–4 each) for 12 h under the indicated conditions. Next, cells in the lower chambers were collected and absolute live cell numbers (#) of MODPs and MOPs **(E)** or MOPs **(F)** were determined by flow cytometry. Data are representative of two independent experiments. Error bars indicate SEM, and unpaired two tailed Student's *t-*test was used for statistical evaluation (*p < 0.05, **p < 0.01, ***p < 0.001). **(G)** Visual representation of the proposed model. Upon i.n. challenge with Flagellin (1), type 2 AECs secrete CCL2 (2) which attracts circulating CCR2^+^ MOPs into the lung (3). Subsequently, TLR5 ligation on locally recruited MOPs by Flagellin (4) promotes MΦ generation from these progenitors (5). Dashed line indicates speculative part of the model.

We next investigated by which chemokines the adoptively transferred MOP cells might be recruited from the blood into the lung upon i.n. Flagellin administration. For monocyte recruitment from blood into tissue, the CCL2-CCR2 axis is very important (5). We focused on this possibility, since our transcriptomic (Supplementary Figure 8A) and flow cytometric (Supplementary Figure 8B, Figure 5A) data showed that CCR2, the receptor for CCL2, is expressed on MOPs, and at a higher level than on MODPs. Moreover, alveolar epithelial cells (AECs) reportedly make CCL2 at steady state and increase CCL2 production in response to Flagellin or other stimuli (33–35). We identified type 2 AECs by flow cytometry in a lung digest, on basis of a CD45^−^MHCII^+^CD31^−^ phenotype (36) (Supplementary Figures 8C,E). These AECs expressed TLR5 (Supplementary Figure 8D) and upregulated CCL2 expression upon stimulation with Flagellin *in vitro* (Figures 5B,C, Supplementary Figures 8E,F) and *in vivo* (Supplementary Figures 8G,H). Also, CCR2 was upregulated on MOPs in the lung after i.n. Flagellin inoculation (Supplementary Figure 7C).

To investigate whether in our *in vivo* setting, CCL2 from Flagellin-treated airway cells may be involved in attracting CCR2^+^ MOPs (or MODPs) into the lung, we performed *in vitro* transwell assays. LLF from mice (see Methods) was placed in the lower chambers of a transwell plate and total BM cells or flow sorted MOPs were plated in the upper chambers (Figure 5D). After 12 h, the MODPs or MOPs in the lower chambers were flow cytometrically determined and quantified (Supplementary Figure 9). In the experiments with total BM cells as input (Figure 5E), both MODPs and MOPs migrated more efficiently toward LLF from mice that had received Flagellin as compared to PBS, with the MOPs being most efficient (Figure 5E). This was confirmed for MOPs, in experiments with purified MOPs as input (Figure 5F). Importantly, in these experiments, CCR2 blockade with specific antibody added to the upper chambers inhibited the migration of MOPs toward LLF from Flagellin-treated mice, but not from PBS-treated mice (Figure 5F). Our collective data suggest that MOP migrate into the lung upon i.n. Flagellin stimulation by virtue of the CCL2-CCR2 axis (Figure 5G).

## Discussion

When a pathogen enters a tissue, epithelial and hematopoietic cells may be activated by PRRs and produce chemokines and cytokines that attract immune cells from blood into tissue (5, 37). In addition, myeloid progenitor cells in the BM may be stimulated to produce monocytes and granulocytes, a process called “emergency myelopoiesis.” In this way, myeloid effector cells that are non-dividing and have a short life span can be replenished (37). The discovery that HSPCs can express PRRs led to two potential mechanisms for emergency myelopoiesis. One possibility is that upon infection serum levels of cytokines such as GM-CSF, G-CSF, and IL-3 increase, which can promote myelopoiesis from progenitors. Alternatively or in addition, myeloid progenitors may respond directly to the pathogen *via* PRRs and give more offspring. PAMPs or serum cytokines do not need enter the BM to reach HSPCs, since these cells circulate between blood and BM at steady state (38). Our study supports a scenario in which bacterial infection at mucosal sites leads to the attraction of myeloid progenitors from the blood into the infected site, where a PAMP (Flagellin) stimulates these progenitors *via* their own PRR (TLR5) to spin off MΦ progeny (Figure 5G).

Upon i.n. administration, Flagellin mainly remains localized in the lung (9). Consistently, we observed in our experiments that Flagellin promoted MΦ differentiation in the lung, but not in blood or BM. Intraperitoneal injection of Flagellin was shown earlier to result in proliferation of myeloid-primed progenitor cells, termed MMP3 cells (Lin^−^Sca-1^+^c-Kit^+^Flt-3^−^CD150^−^CD48^+^) (22), which are distinct from MO(D)Ps. It was not examined whether these cells expressed TLR5. To our knowledge, we are the first to report TLR5 expression on hematopoietic progenitors. Flagellin promoted MΦ differentiation from MO(D)Ps, but not OC differentiation. Flagellin has been reported to promote OC formation in assays with whole bones, but in agreement with our findings, it did not affect RANKL/M-CSF-induced OC differentiation from progenitors (39).

In our *in vivo* setting, we transferred MOPs i.v. to mimic the physiological situation, in which progenitors circulate between blood and BM (38). We could not trace the adoptively transferred MOPs back to the lung, but we did detect their MΦ offspring. In addition, we detected recruitment of endogenous MOPs of the recipient mice into the lung in response to Flagellin inoculation. We considered that MOPs might be attracted from the blood into the lung by CCL2, because lung epithelial cells secrete CCL2 in response to Flagellin (33, 34) and CCL2 is important to attract monocytes into tissue (5). Also, CCR2 is important for the trafficking of hematopoietic cells to sites of inflammation (40). CCR2 expression was higher on the MΦ/OC committed MOP than on the upstream MODP, which is in line with earlier findings that CCR2 expression on HSPCs progressively increases from self-renewing HSCs, to MPPs, to specialized myeloid progenitors CMPs and GMPs (40). *In vitro*, type 2 AECs produced more CCL2 upon stimulation with Flagellin. We also found that LLF from Flagellin-treated mice attracted more MOPs than did LLF from PBS-treated mice, which relied on CCR2. These data suggest that CCL2 from Flagellin-stimulated AECs led to the recruitment of MOPs into the lung (Figure 5G). This possibility can be further addressed by *in vivo* blockade of the CCL/CCR2 axis.

Interestingly, it has also been reported that hematopoietic progenitor cells can migrate at steady state from blood into a diversity of peripheral tissues (41). Accordingly, we found endogenous MOPs in lungs of CD45.2 recipient mice in absence of Flagellin inoculation. Therefore, the presence of PRRs on these progenitors may enable them to directly sense pathogens in infected tissue and spin off progeny locally when the appropriate differentiation factors are present. This may be the earliest effect on progenitors during infection and may be followed by progressive recruitment of progenitors into the tissue for amplification of the response.

In summary, we here provide evidence for a scenario in which Flagellin-sensing by lung epithelial cells leads to a rapid influx of MΦ/OC progenitors from the circulation and Flagellin sensing by these progenitors promotes local MΦ differentiation. We suggest that this type of emergency myelopoiesis enables quick and qualified phagocyte replenishment, representing another layer of host protection against bacterial infections at mucosal sites. Future studies should address whether TLR5-mediated MΦ differentiation from myeloid progenitors promotes host protection against bacterial infection.

## Data Availability Statement

The datasets presented in this study can be found in online repositories. The name of the repository and accession number can be found here: NCBI (accession: GSE97380).

## Ethics Statement

The animal study was reviewed and approved by the IVD of the Netherlands Cancer Institute.

## Author Contributions

YX designed the research, designed and performed animal experiments, analyzed and interpreted data, and wrote the manuscript. JB designed the research, interpreted data, and wrote the manuscript. XL and JP designed and performed experiments, analyzed data, and wrote the manuscript. TW contributed to experiments. MB advised on flow cytometry and contributed to cell sorting. IR contributed to data analysis. All authors contributed to the article and approved the submitted version.

## Conflict of Interest

The authors declare that the research was conducted in the absence of any commercial or financial relationships that could be construed as a potential conflict of interest.
