# Supplementary material for: Flagellin/TLR5 Stimulate Myeloid Progenitors to Enter Lung Tissue and to Locally Differentiate Into Macrophages
Source: Front Immunol. 2021 Mar 19;12:621665. doi: 10.3389/fimmu.2021.621665 (PMC8017192; doi:10.3389/fimmu.2021.621665)
Supplement: Supplementary file 1 [file Data_Sheet_1.pdf]

Supplementary information

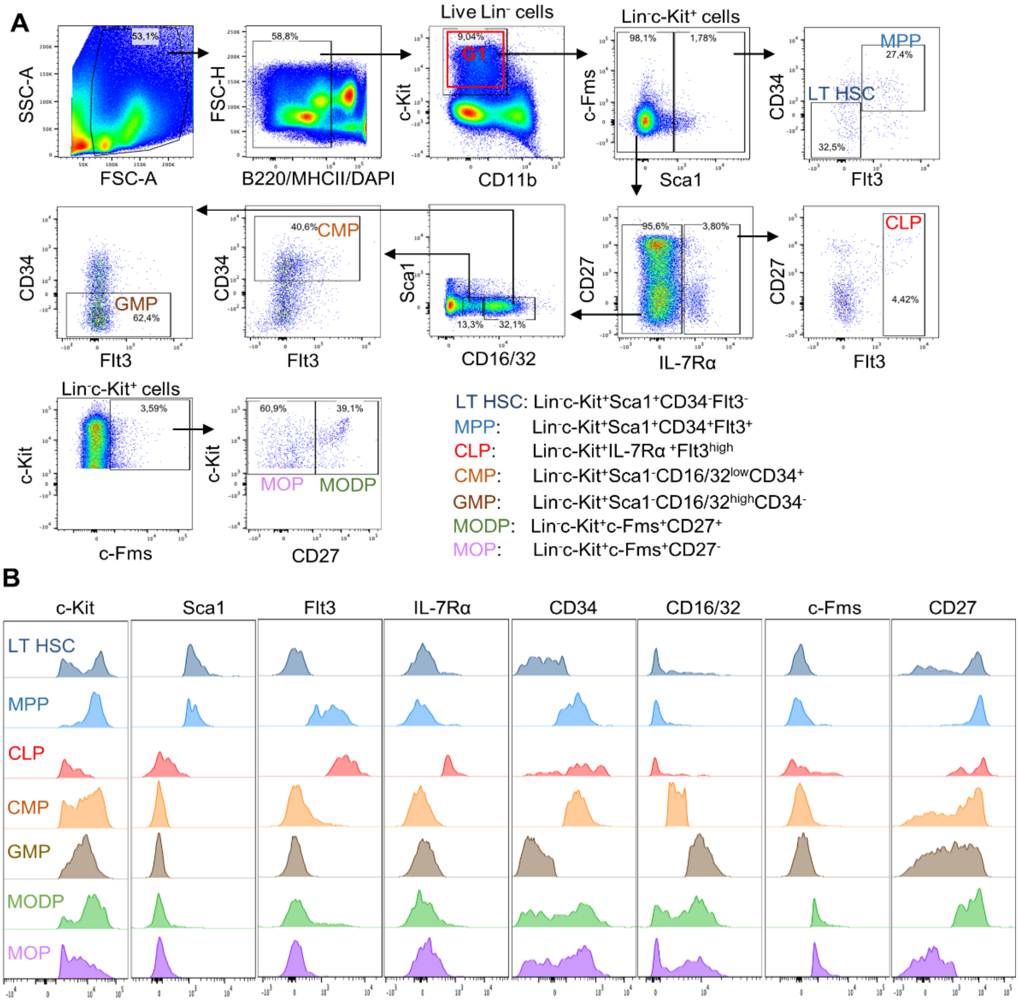

C

	LT-HSC	MPP	CLP	CMP	GMP	MODP	MOP
c-Kit	+	+	+ (low)	+	+	+	+ (low)
Sca1	+	+	low/-	-	-	-	-
Flt3	-	+	high	-	-	-/+	-
IL-7Rα	-	-	+	-	-	-	-
CD34	-	+	+/-	+	-	+/-	+/-
CD16/32	-	-	-	low	high	+/-	+/-
c-Fms	-	-	-/+	-	-	+	+
CD27	+/-	+	+	+/-	+/-	+	-

Figure S1. Cell surface phenotype of MODPs, MOPs and other progenitors. BM cells were simultaneously stained with antibodies detecting MODP and MOP and all characteristic progenitor markers indicated. **A**) Gating strategy of live (DAPI$^-$) B220$^-$MHCII$^-$CD11b$^{low/-}$c-Kit$^+$ mouse BM cells (G1) and its further separation into long term (LT)-HSC, MPP, CLP, CMP, GMP as defined by the indicated cell surface markers defined in ref. 30, as well as MODP and MOP as defined in ref. 26. (**B**) Histograms depicting the cell surface expression of the indicated markers on the indicated progenitor populations. (**C**) Overview of the defining markers for the indicated progenitor populations according to refs. 26 and 30 (black) and according to this flow cytometric analysis (red). Data are representative of 2 experiments with 2 mice each.

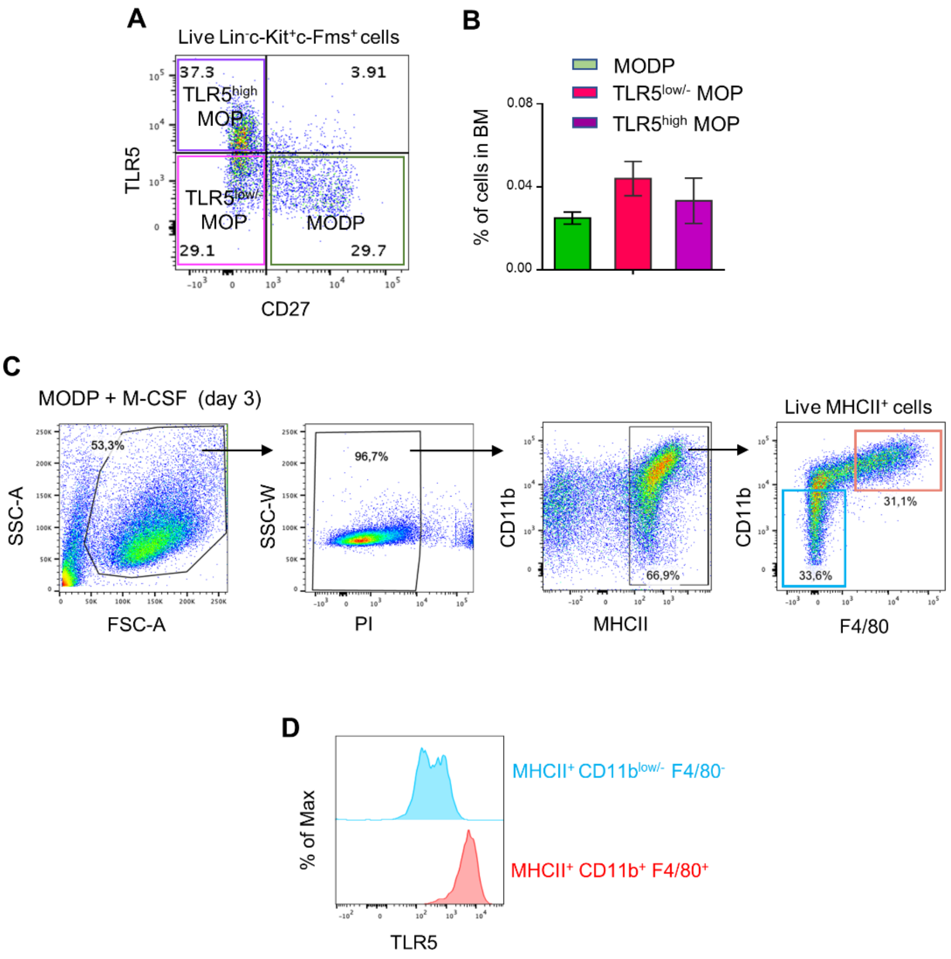

Figure S2. Identification of TLR5$^{low/-}$and TLR5^{high} MOPs in BM and TLR5 detection on offspring of the MODP in MΦ differentiation culture. (**A**) Representative flow cytometry plot depicting the gating strategy to distinguish MODPs, TLR5$^{low/-}$ and TLR5^{high} MOPs in live Lin^{-}c-Kit^{+}c-Fms^{+} BM cells. (**B**) Bar chart depicting the frequencies (%) of MODPs, TLR5$^{low/-}$ and TLR5^{high} MOPs among total live cells in BM, as diagnosed by flow cytometry. Data is pooled from three independent experiments with technical duplicates of pooled BM cells from two mice (error bars indicate SD). (**C-D**) MODPs were seeded at 2,000 cells/well and cultured with M-CSF. After 3 days, offspring cells were analyzed by flow cytometry. (**C**) Representative flow cytometry plots depicting the gating strategy for MHCII^{+}CD11b^{+}F4/80^{+} and MHCII^{+}CD11b$^{low/-}$F4/80^{-} cell populations in the culture. (**D**) Histogram depicting TLR5 cell surface expression on the indicated populations. Data are representative of three independent experiments. In each experiment, MODPs were sorted from pooled BM cells from 3-4 mice and technical duplicates were used for differentiation cultures.

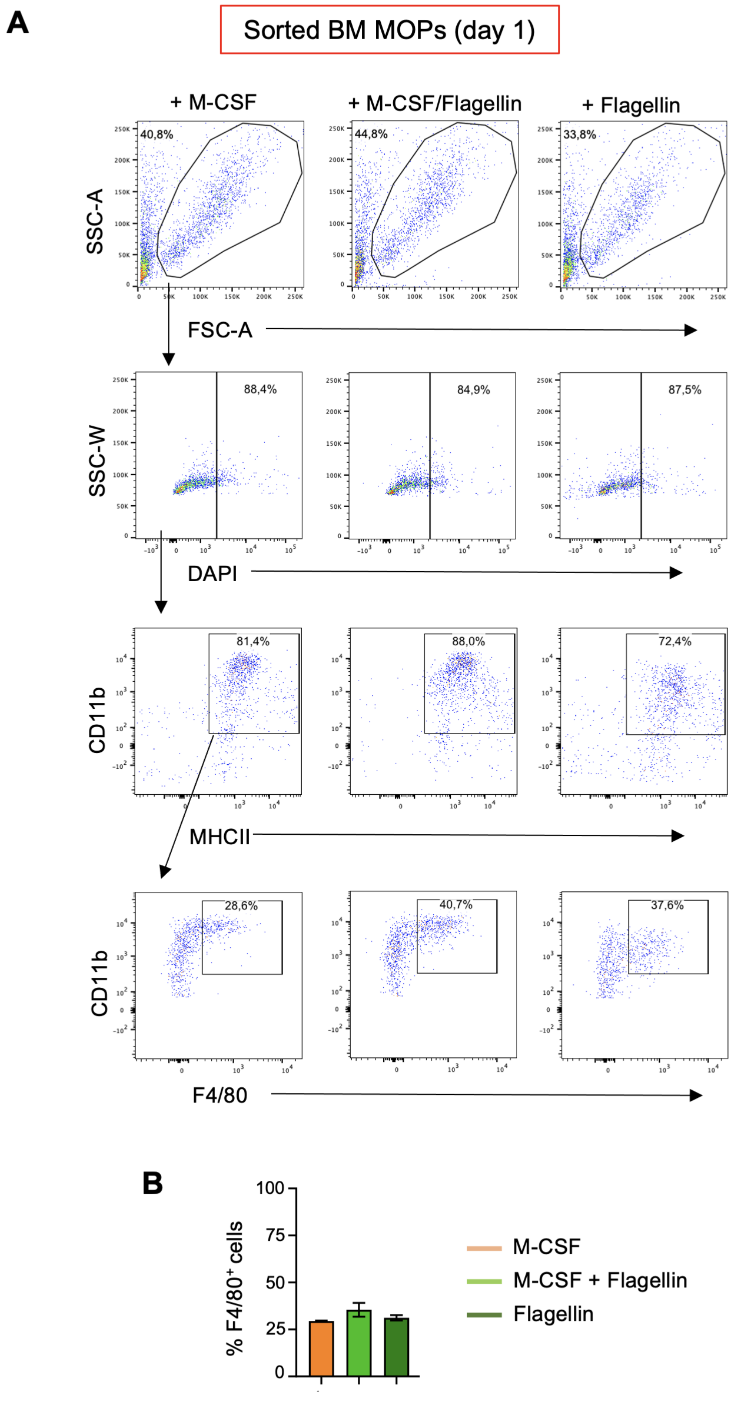

Figure S3. Gating strategy and quantification of MOP offspring cells in *in vitro* MΦ differentiation culture at day 1. MOPs were seeded at 2,000 cells/well and cultured with M-CSF with or without Flagellin, or with Flagellin only. Offspring cells were analyzed by flow cytometry after 1 day of culture. (**A**) Representative flow cytometry plots depicting gating strategy for MΦs, defined as $MHCII^+CD11b^+F4/80^+$ cells. (**B**) Bar chart depicting frequencies (%) of $F4/80^+$ cells in the $MHCII^+CD11b^+$ gate. Data are representative of two independent experiments, in which MOPs were sorted from pooled BM cells from 3-4 mice and technical duplicates were used for differentiation cultures. Error bars indicate SEM.

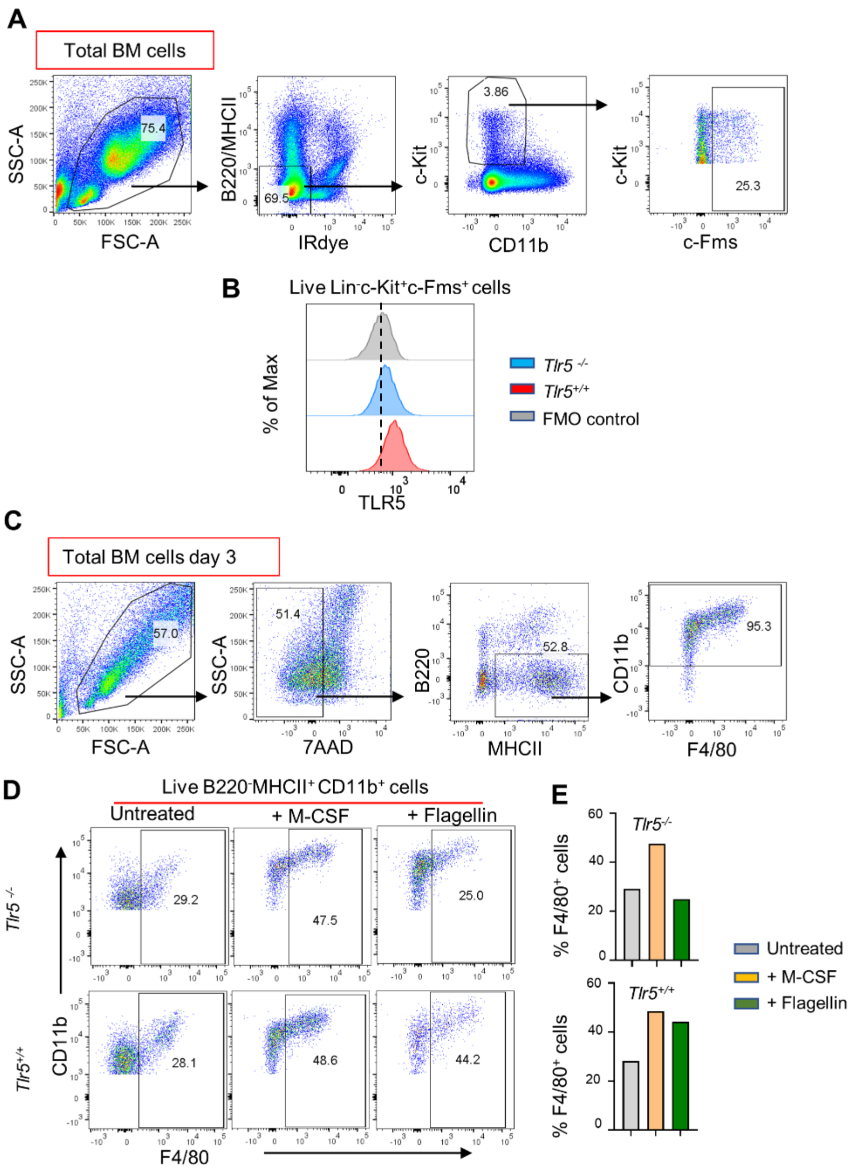

Figure S4. TLR5 staining and MΦ differentiation assay of *Tlr5*$^{-/-}$ and *Tlr5*$^{+/+}$ BM cells. (A-B) BM cells from *Tlr5*$^{-/-}$ and *Tlr5*$^{+/+}$ mice were stained for TLR5 in combination with progenitor markers and analyzed by flow cytometry. (**A**) Representative flow cytometry plots depicting gating strategy for Lin$^-$c-Kit$^+$c-Fms$^+$ cells. (**B**) Histograms depicting TLR5 cell surface staining on Lin$^-$c-Kit$^+$c-Fms$^+$ cells from *Tlr5*$^{-/-}$ (n=1) and *Tlr5*$^{+/+}$ (n=1) BM samples. TLR5 FMO control was from *Tlr5*$^{+/+}$ BM sample. Data is from one experiment with technical duplicates. (**C-E**) Total *Tlr5*$^{-/-}$ and *Tlr5*$^{+/+}$ BM cells were plated at density of 200,000 cells/well and cultured with M-CSF or Flagellin or medium only (untreated) and 3 days later the cultures were analyzed by flow cytometry. (**C-D**) Representative flow cytometry plots depicting the gating strategy for MΦs, defined as MHCII$^+$CD11b$^+$F4/80$^+$ cells. (**E**) Frequencies (%) of F4/80$^+$ cells within live B220$^-$MHCII$^+$CD11b$^+$ cells in the MΦ cultures from *Tlr5*$^{-/-}$ and *Tlr5*$^{+/+}$ BM cells.

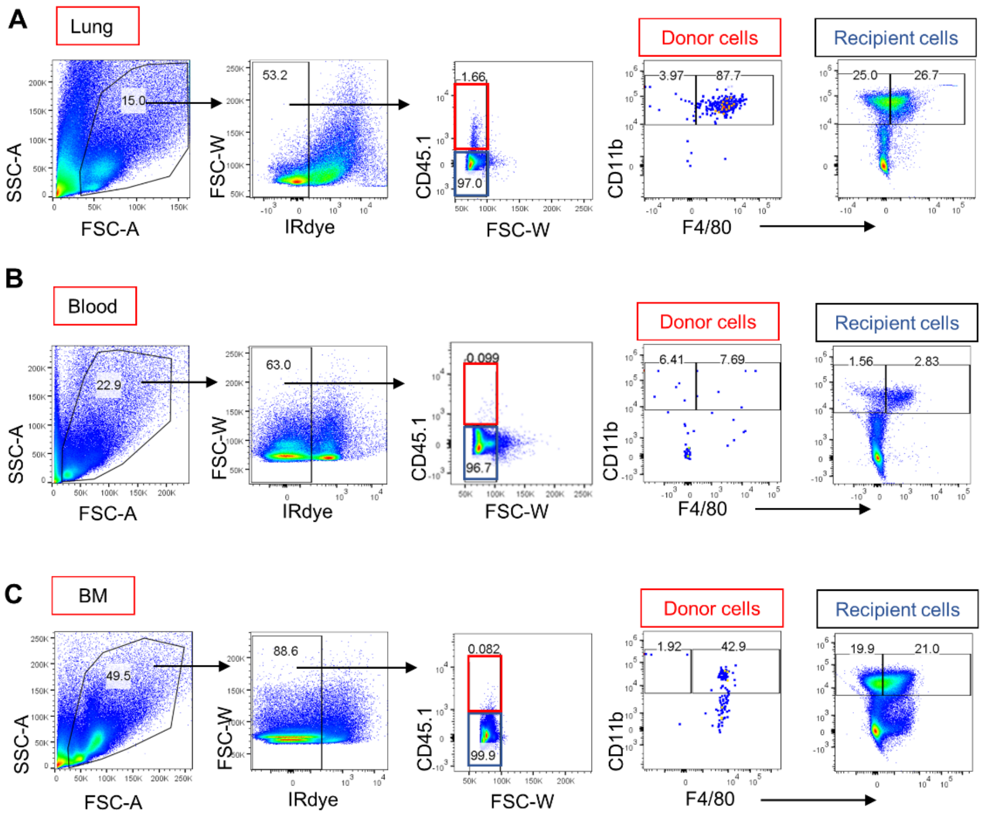

Figure S5. Gating strategy for MΦ from i.v. transferred CD45.1^{+} donor cells CD45.1^{-} recipient cells. MOPs (Lin^{-}c-Kit^{+}c-Fms^{+}CD27$^{low/-}$) were flow cytometrically sorted from pooled BM cells of CD45.1 donor mice (n=5) and intravenously (i.v.) transferred into CD45.2 recipient mice. At 24 h after adoptive cell transfer, 1 µg Flagellin in 50 µl PBS or 50 µl PBS was inoculated intranasally (i.n.) into the recipient mice. 24 hours after i.n. inoculation, lung, blood and BM were harvested and dissociated into single cells for flow cytometric analysis (as described in Figure 4). (**A-C**) Representative flow cytometry plots depicting gating strategy for MΦ, defined as MHCII^{+}CD11b^{+}F4/80^{+} cells among CD45.1^{+} donor cells and CD45.1^{-} recipient cells in the lung, blood and BM respectively. Data are representative of two independent experiments each with n=4 recipient mice per group.

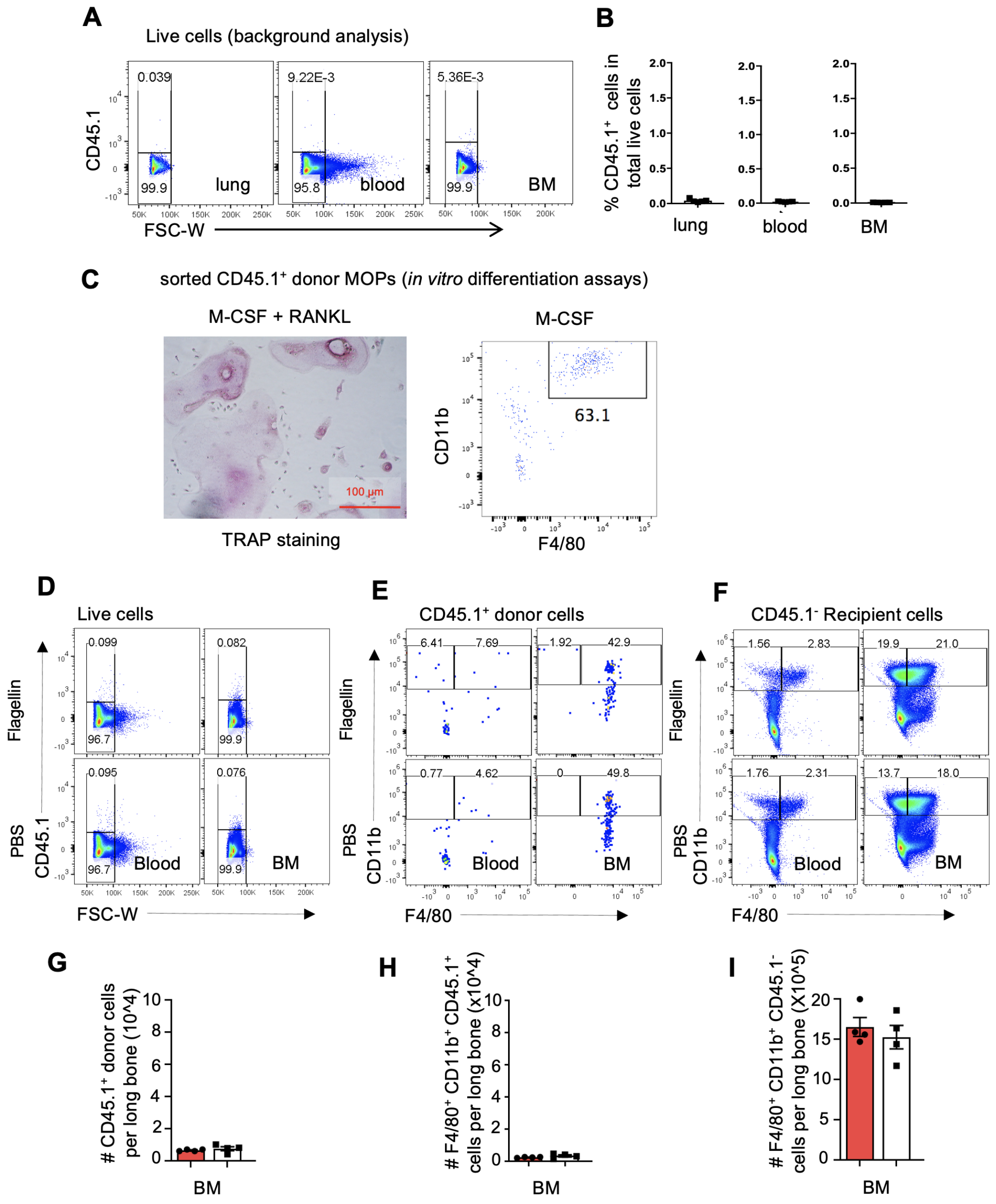

Figure S6. MΦ differentiation from i.v. transferred MOPs in blood and BM. (**A**) CD45.2 mice that did not receive cells or treatment were used for background analysis. Representative flow cytometry plots depicting frequencies (%) of cells staining with antibody to CD45.1 in lung, blood and BM. (**B**) Quantification of background CD45.1 staining. Data is from one experiment with 3 mice. (**C**) *In vitro* evaluation at day 5 of OC and MΦ differentiation potential of CD45.1$^+$ MOPs prior to adoptive transfer. Light microscopic image (left) depicting TRAP-stained cells in OC culture and flow cytometry plot (right) depicting frequencies (%) of MΦs, defined as MHCII$^+$CD11b$^+$F4/80$^+$ cells in the MΦ culture. (**D-F**) Representative flow cytometry plots depicting adoptively transferred CD45.1$^+$ cells, CD45.1$^+$ MOP-derived MΦs and CD45.1$^-$ endogenous MΦs in blood and BM. (**G-I**) Quantification of CD45.1$^+$ cells, CD45.1$^+$ MOP-derived MΦs and CD45.1$^-$ endogenous MΦs in BM, as indicated by absolute numbers (#) per long bone. Data are representative of two independent experiments with n=4 each. Error bars indicate SEM, and unpaired two tailed Student's *t* test was used for statistical evaluation.

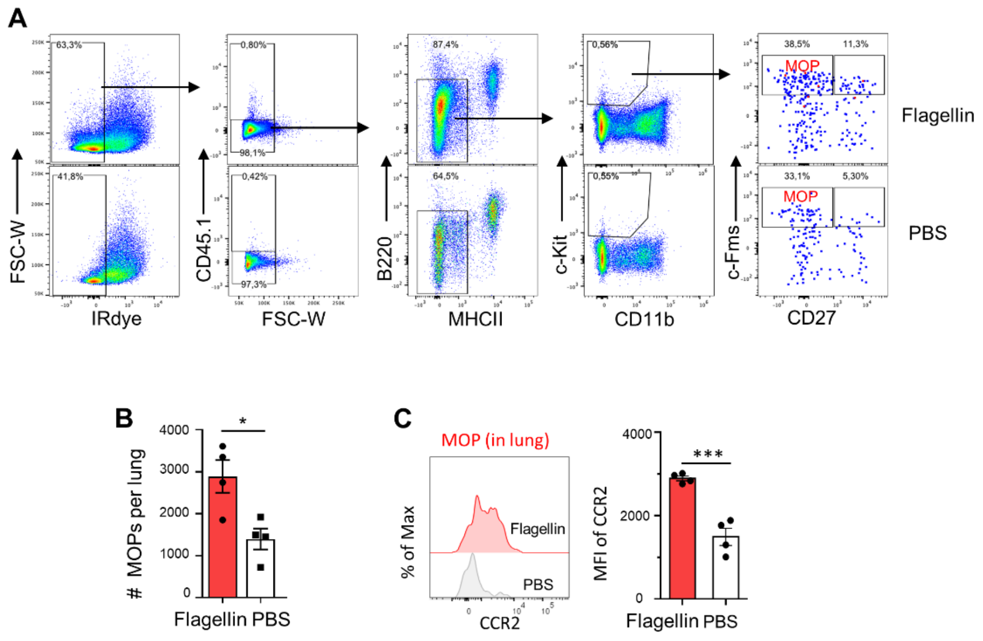

Figure S7. Detection of CD45.1⁻ recipient MOPs and their CCR2 expression in the lung. (A-D) Lung single-cell suspensions from mice that had been inoculated i.n. with Flagellin or PBS (as described in Figure 4) were stained with antibody to CCR2 in combination with antibodies to MOP markers and analyzed by flow cytometry. (**A**) Representative flow cytometry plots depicting gating strategy for CD45.1$^-$ MOPs in the lung from mice that had received i.n. Flagellin or PBS. (**B**) Quantification of CD45.1$^-$ MOPs in the lung, as indicated by absolute numbers (#). (**C**) Cell surface detection of CCR2 by flow cytometry on MOPs in the lung from mice that had received i.n. Flagellin or PBS as indicated by histogram (left) and MFI (right). Data are representative of two independent experiments with n=4 for S7A, B. Data are from one experiment with n=4 for S7C. Error bars indicate SEM, and unpaired two tailed Student's *t* test was used for statistical evaluation.

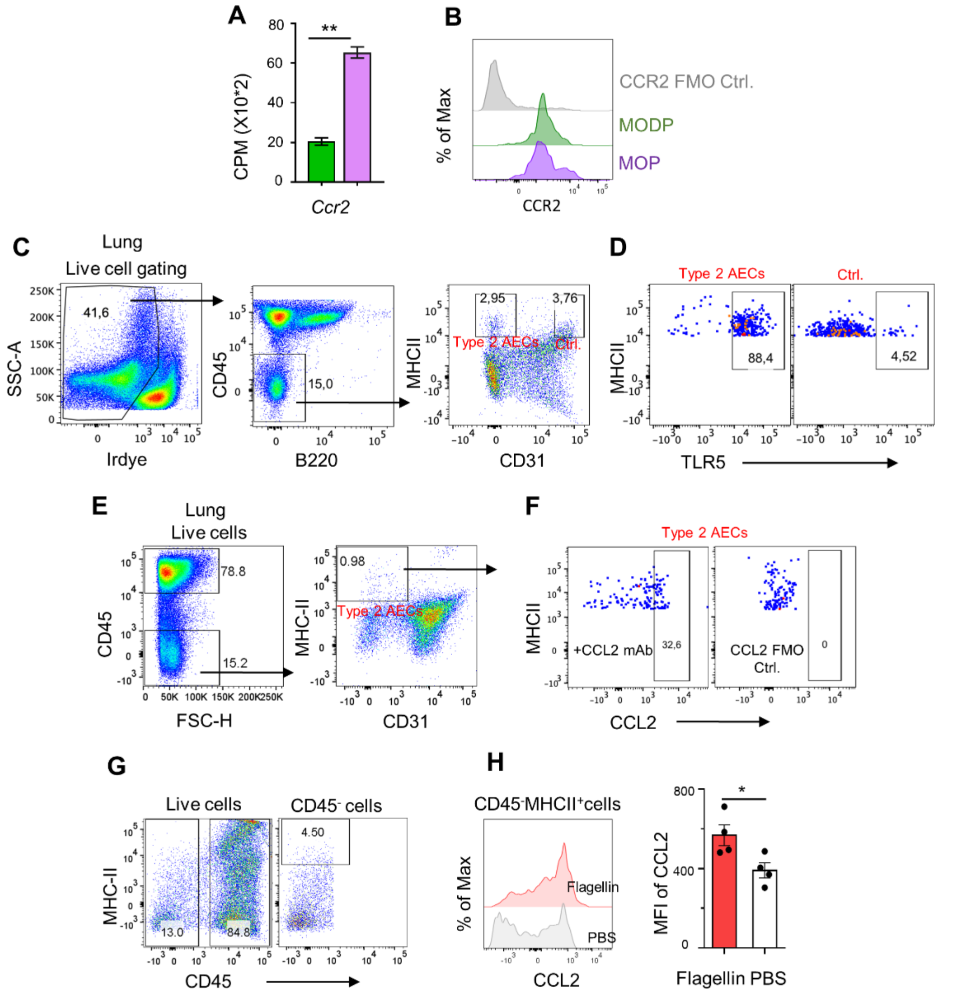

Figure S8. Detection of CCR2 in MODPs and MOPs in BM at transcript and protein level, and detection of CCL2 and TLR5 in type 2 AECs in lung. (**A**) *Ccr2* mRNA levels in MODPs and MOPs based on normalized read counts (CPM) (n=3), as determined by transcriptomics (GSE97380;(26)) (see legend to Figure 1D). **(B)** Histogram depicting flow cytometric detection of CCR2 on MODP and MOP populations in mouse BM. CCR2 FMO was used as control. Data are representative of two independent experiments with n=2. (**C**, **D**) Single-cell suspensions of lung tissue were stained to detect TLR5 and type 2 AECs by flow cytometry. Representative flow cytometry plots depicting gating strategy for live type 2 AECs defined as CD45^{-}MHCII^{+}CD31^{-} cells (**C**) and cell surface expression of TLR5 on type 2 AECs (**D**). CD45^{-}MHCII^{+}CD31^{+} cells were used as controls (Ctrl.). **(E, F)** Single-cell suspensions of lung tissue were cultured in medium with or without Flagellin overnight, stained for CCL2 (intracellularly) and type 2 AEC markers and analyzed by flow cytometry. Flow cytometry plots depicting gating strategy for live type 2 AECs defined as CD45^{-}MHCII^{+}CD31^{-}cells (**E**) and intracellular CCL2 expression in type 2 AECs (**F**). CCL2 FMO was used as control. For statistical information for panels C-F, see Figure 5B. (**G**,**H**) Lung single-cell suspensions from mice that had been inoculated i.n. with Flagellin or PBS (as described in Figure 4) were stained with antibody to CCL2 (intracellular) in combination with antibodies to CD45 and MHCII, and analyzed by flow cytometry. (**G**) Representative flow cytometry plots depicting gating strategy for CD45^{-}MHCII^{+} non-hematopoietic cells in the lung tissues. (**H**) Plots depicting CCL2 detection by intracellular staining in CD45^{-}MHCII^{+} non-hematopoietic cells in histogram (left) and MFI (right). CCL2 FMO was used as control. MFI was calculated as median value of the fluorescent staining. For statistical information for panels G, H, see Figure 4.

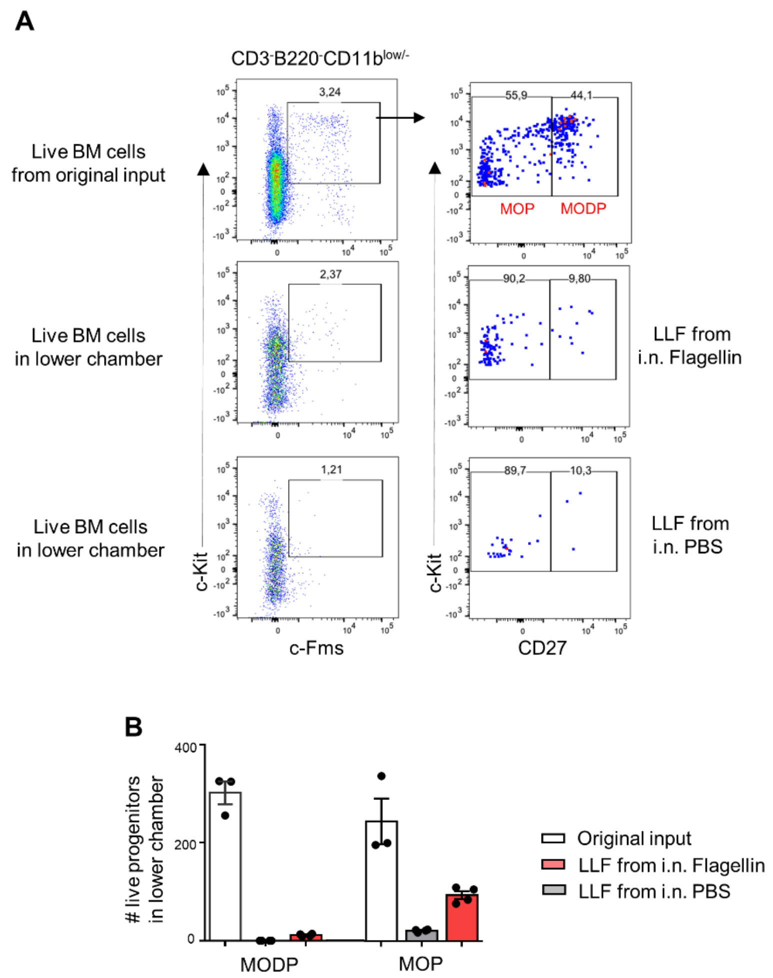

Figure S9. Detection of MODP and MOP migration. LLF was harvested as described in Materials and Methods from the two groups of mice that had been used for the *in vivo* experiments. LLF was placed in the lower chambers of transwell plates. In the upper chambers, pooled BM cells from three mice were seeded at density of 100,000 cells per well. 100,000 total BM cells per well were directly placed in the lower chambers of transwell plates to obtain the original input information. After 12 h of incubation, cells in the lower chambers were collected for phenotyping and quantification by flow cytometry. **(A)** Representative flow cytometry plots depicting the gating strategy for B220^{-}CD11b$^{low/-}$c-Kit^{+}c-Fms^{+} CD27^{high} MODPs and CD27$^{low/-}$ MOPs in the lower chambers. (**B**) Bar chart depicting total live cell number (#) of MODPs and MOPs as original input and total live cell numbers of MODPs and MOPs that migrated into the lower chambers. Data are representative of two independent experiments and each experiment includes LLF from n=3-4. Error bars indicate SEM.

Supplemental Table 1.

Antibody and reagent list

Antibody	*Fluorochrome*	*Clone*	*Manufacturer*	*Dilution*	*Catalogue*
CD3 #	AF488	17A2	BioLegend	1:100	100210
CD11b *#	BV786	M1/70	BioLegend	1:100	101243
CD16/32 #	BV510	93	BioLegend	1:100	101333
CD27 *#	PE	LG.3A10	BD Biosciences	1:100	558754
CD27 *#	PE-Dazzle 594	LG.3A10	BioLegend	1:100	124228
CD27 #	FITC	LG.3A10	home made	1:100	n/a
CD31 #	PE	390	BioLegend	1:100	102408
CD34 #	AF700	RAM34	eBioscience	1:100	56-0341-82
CD45 #	BV605	30-F11	BioLegend	1:50	103139
CD45.1 #	BV650	A20	BioLegend	1:75	110735
c-Fms/ CD115 *	APC	AFS98	BioLegend	1:100	135510
c-Fms/ CD115 *#	BV711	AFS98	BioLegend	1:100	135515
c-Kit/ CD117 *#	PE-Cy7	2B8	eBioscience	1:100	25-1171-82
IL7Rα/ CD127 #	BV650	A7R34	BioLegend	1:100	135043
Flt3/ CD135 #	PE	A2F10	BD Biosciences	1:75	121351
B220 *#	FITC	RA3-6B2	BioLegend	1:100	103206
B220 *#	Percp-cy5.5	RA3-6B2	BioLegend	1:75	103236
B220 #	PB	RA3-6B2	BD Biosciences	1:100	558108
CCR2 #	PE	SA203G11	BioLegend	1:100	150610
CCL2 #	APC	2H5	BioLegend	1:50	505910
F4/80 #	APC-efluor780	BM8	eBioscience	1:100	47-4801-82
F4/80 #	AF700	BM8	eBioscience	1:50	56-4801-82
MHCII/ I-A/I-E #	AF700	M5/114.15.2	BioLegend	1:100	107622
MHCII/ I-A/I-E #	PE/Dazzle 594	M5/114.15.2	BioLegend	1:200	107648
MHCII/ I-A/I-E #	FITC	2G9	BD Biosciences	1:100	553623
MHCII/ I-A/I-E #	PB	M5/114.15.2	BioLegend	1:100	107620
RANK #	PE	R12-31	eBioscience	1:100	12-6612-82
Sca-1/ Ly-6A/E #	Percp-cy5.5	D7	eBioscience	1:100	45-5981-82
TLR5 *#	AF405	85B152.5	NOVUSBIO	1:50	NBP1-97728AF405
DAPI		N/A	Sigma	1*ug*/ml	MBD0015
PI		N/A	BioLegend	1*ug/ml*	421301
7-AAD		N/A	BioLegend	1:20	420403
LIVE/DEAD™ Fixable Near-IR Dead Cell Stain Kit #		N/A	Invitrogen	1:1000	L34976

Antibodies used only for progenitor sorting are marked with *, antibodies only used for cell phenotyping are marked with # and antibodies used for both purposes are marked with *#.
